# Purification and Characterization of a Bifunctional Alginate Lyase from *Pseudoalteromonas* sp. SM0524

**DOI:** 10.3390/md9010109

**Published:** 2011-01-21

**Authors:** Jian-Wei Li, Sheng Dong, Jie Song, Chun-Bo Li, Xiu-Lan Chen, Bin-Bin Xie, Yu-Zhong Zhang

**Affiliations:** 1 State Key Laboratory of Microbial Technology, Marine Biotechnology Research Center, Shandong University, Jinan 250100, China; E-Mails: sdnyljw@163.com (J.-W.L.); Ds518@126.com (S.D.); songjie_63@hotmail.com (J.S.); xbb@sdu.edu.cn (B.-B.X.); zhangyz@sdu.edu.cn (Y.-Z.Z.); 2 Biomedical Analysis Center, Tsinghua University, Beijing 100084, China; E-Mail: lichunbo@biomed.tsinghua.edu.cn (C.-B.L.)

**Keywords:** bifunctional alginate lyase, *Pseudoalteromonas* sp. SM0524, alginate

## Abstract

An alginate lyase-producing bacterial strain, *Pseudoalteromonas* sp. SM0524, was screened from marine rotten kelp. In an optimized condition, the production of alginate lyase from *Pseudoalteromonas* sp. SM0524 reached 62.6 U/mL, suggesting that strain SM0524 is a good producer of alginate lyases. The bifunctional alginate lyase aly-SJ02 secreted by strain SM0524 was purified. Aly-SJ02 had an apparent molecular mass of 32 kDa. The optimal temperature and pH of aly-SJ02 toward sodium alginate was 50 °C and 8.5, respectively. The half life period of aly-SJ02 was 41 min at 40 °C and 20 min at 50 °C. Aly-SJ02 was most stable at pH 8.0. N-terminal sequence analysis suggested that aly-SJ02 may be an alginate lyase of polysaccharide lyase family 18. Aly-SJ02 showed activities toward both polyG (α-l-guluronic acid) and polyM (β-d-mannuronic acid), indicating that it is a bifunctional alginate lyase. Aly-SJ02 had lower *K*_m_ toward polyG than toward polyM and sodium alginate. Thin layer chromatography and ESI-MS analyses showed that aly-SJ02 mainly released dimers and trimers from polyM and alginate, and trimers and tetramers from polyG, which suggests that aly-SJ02 may be a good tool to produce dimers and trimers from alginate.

## 1. Introduction

Alginate is a gelling polysaccharide found in great abundance as part of the cell wall and intracellular material in brown seaweeds (Phaeophyceae) [1]. Alginate is a linear hetero-polyuronic acid composed of 1,4 linked α-l-guluronic acid (G) and β-d-mannuronic acid (M). These two residues are arranged in block structures comprising homopolymeric G blocks, M blocks, alternating MG (GM) blocks, and heteropolymeric MG (GM) blocks [2]. Alginate is widely used as a stabilizer, viscosifier, and gelling agent in the food and beverage, paper and printing, biomaterials, and pharmaceutical industries.

Alginate lyases, also known as alginases or alginate depolymerases, catalyze the degradation of alginate by a *β*-elimination mechanism that has yet to be fully elucidated [1]. Alginate lyases are isolated from various sources, such as marine algae, marine mollusks, fungi, bacteria and viruses. Alginate lyases are classified into three groups by their substrate specificity toward G blocks (polyguluronate lyase; EC 4.2.2.11), M blocks (polymannuronate lyase; EC 4.2.2.3), or MG blocks [1]. Those specific to G blocks or M blocks are called monofunctional alginate lyases, and those specific to MG blocks are called bifunctional alginate lyases [3]. Furthermore, they are also grouped into three types based on their molecular masses: small (25–30 kDa), medium-sized (around 40 kDa), and large lyases (>60 kDa) [4]. Alginate lyases have a wide variety of uses. They are used in the production of algal protoplasts and for studying the fine structure of alginate. They have also been used as tools for generating useful oligomeric products of alginate that can be used as therapeutic agents, physiological food sources or plant growth promoters [1]. With the discovery and characterization of novel alginate lyases, further applications in biotechnology may be found.

In this study, an alginate lyase-producing bacterium, *Pseudoalteromonas* sp. SM0524, was screened from marine rotten kelp. The bifunctional alginate lyase aly-SJ02 secreted by this strain was purified and characterized, and its action on alginate was analyzed.

## 2. Experimental Section

### 2.1. Materials

Sodium alginate from brown algae was purchased from Sigma (USA). PolyM and PolyG (purity: about 95%) were kindly provided by Professor Wengong Yu in Ocean University of China.

### 2.2. Screening and identification of strain SM0524

The rotten kelp was collected from a kelp culture field at the seashore of Yantai, China, in May, 2005. The rotten kelp was cut into small pieces. A 500-mL flask containing 200 mL enrichment medium (0.5% peptone, 0.1% yeast extract, 0.5% sodium alginate, 3% NaCl, pH 6.5) and 5 g kelp pieces were incubated at 180 rpm, 25 °C for 24 h to enrich alginate lyase-producing bacteria. After enrichment, the culture was serially 10-fold diluted to 10^−6^ dilution with sterile seawater. Aliquots of 100 μL diluted samples (10^−1^–10^−6^ dilution ) were spread on screening plates with a medium composed of 1% sodium alginate, 0.5% (NH_4_)_2_SO_4_, 0.2% K_2_HPO_4_·3H_2_O, 0.001% FeSO_4_·7H_2_O, 0.1% MgSO_4_·7H_2_O, 3% NaCl, 1.5% agar (pH 7.5). The plates were then incubated at 25 °C for 1~3 d to form detectable colonies. One hundred strains were selected from the screening plates and purified by repeated streaking on the same medium. The purified strains were stippled on screening plates, respectively, and inoculated at 25 °C for 2 d. Lugol solution (5 mL) was spread on each screening plate to show the clear hydrolytic zone around a strain as described by Schlesner *et al.* [5]. Ten strains with relatively big hydrolytic zones were inoculated into a liquid medium with the same composition as the enrichment medium and cultured at 180 rpm, 25 °C to detect their ability to reduce the viscosity of the medium. After 44 h cultivation, the alginate lyase activity in the culture was measured.

The 16S rRNA gene of SM0524 was amplified by PCR from the genomic DNA and sequenced as described by Hu and Li [6]. The obtained 16S rRNA gene sequence was aligned using CLUSTAL X (v 1.83) with its closely related sequences retrieved from GenBank. The 16S rRNA gene sequence of SM0524 was deposited in GenBank under the accession number EU548075.

### 2.3. Production and purification of the alginate lyase aly-SJ02

Strain SM0524 was inoculated in 150 mL optimized liquid medium containing 0.5% peptone, 0.1% yeast extract, 0.2% sodium alginate, 2.5% NaCl (pH 7.0) in a 500 mL flask and incubated on a rotary shaker (180 rpm) at 15 °C for 60 h to reach the highest alginate lyase activity in the culture. Then, the culture was centrifuged at 10,000×g, 4 °C for 5 min. The supernatant was concentrated by ultrafiltration with a membrane (molecular weight cut-off 3 kDa) and then was dialyzed against 40 mM phosphate buffer (pH 6.0). The enzyme solution was purified by anion-exchange chromatography on a DEAE Sepharose Fast Flow column which had been pre-equilibrated with 40 mM phosphate buffer (pH 6.0). Proteins were eluted with a linear gradient of 0.1 to 0.5 M NaCl at a flow rate of 1 mL/min. The fractions with alginate lyase activity were further purified by gel filtration on a Sephadex G-100 column, which was eluted with 40 mM phosphate buffer (pH 6.0). The fractions with alginate lyase activity were collected. Its purity and molecular mass was determined with a sodium dodecyl sulfatepolyacrylamide gel electrophoresis (SDS-PAGE) system (Bio-Rad) as described by Laemmli [7].

### 2.4. Protein determination and enzyme assay

The alginate lyase activity of aly-SJ02 was measured by Somogyi’s method [8]. Briefly, a reaction mixture containing 20 μL properly diluted enzyme, 80 μL 50 mM Tris-HCl buffer with 0.25 mM NaCl (pH 8.5) and 100 μL 0.5% sodium alginate was indicated at 50 °C for 30 min. After incubation, 200 μL alkaline copper reagent was added to stop the reaction and the reducing sugar released in the mixture was determined with mannose as a standard. One unit of enzyme activity was defined as the amount of enzyme used for the production of 1 μmol of reducing sugar per min. The alginate lyase activity of aly-SJ02 was ascertained by measuring the absorbance of the generated products at 235 nm according to Gacesa [9] in analyzing the kinetic constants. Protein concentration was determined using Bradford’s method [10].

### 2.5. Characterization of the alginate lyase aly-SJ02

To determine the optimal temperature, the activity of the purified alginate lyase aly-SJ02 toward sodium alginate was assayed at pH 8.5 in a range of 3–65 °C. To determine the optimal pH, the alginate lyase activity of aly-SJ02 was measured at 50 °C in the range of pH 5.0–10.0 in a broad buffer as described previously [11]. The thermal stability of aly-SJ02 was determined by measuring the residual activity of the enzyme after incubating at 30 °C, 40 °C, and 50 °C for different periods of time. The pH stability of aly-SJ02 was determined measuring the residue activity of the enzyme after incubating the enzyme in the broad pH buffer from pH 4.0 to pH 11.0 at 25 °C for 20 min. To investigate the effect of NaCl on the activity of aly-SJ02, 0–1.2 M NaCl was added to the reaction mixture, and then the enzyme activity was assayed with sodium alginate as substrate. To assay the effects of metal ions and EDTA on the activity of aly-SJ02, each metal ion (or EDTA) was incubated with aly-SJ02 at 0 °C for 30 min, and then the activity of aly-SJ02 toward sodium alginate was measured. The reaction mixture without any metal ion or inhibitor was taken as control. The *K*_m_ values of aly-SJ02 toward sodium alginate, polyM and polyG were determined by non-linear analysis based on the initial rates determined with 0.3–6 mg/mL of each substrate at 50 °C. To determine the N-terminal sequence of aly-SJ02, The purified aly-SJ02 was electrophoresed into SDS-PAGE gel, and then was transferred to a Sequi-Blot polyvinylidene difluoride membrane (Bio-Rad, U.S.). The N-terminal sequence of aly-SJ02 was analyzed by Edman degradation with PROCISE491 (Applied Biosystems, U.S.) at Beijing University (China). The sequences for alignment were cited from GenBank and PDB databases. Sequences were aligned by using Clustal X 1.83 [12].

### 2.6. Hydrolysis of polyM, polyG and sodium alginate by aly-SJ02

A 500-μL reaction mixture containing 1 μg aly-SJ02 and 2.5 mg polyM, polyG or sodium alginate was incubated at 40 °C for 2 h. After incubation, the mixture solution was boiled for 5 min and then centrifuged at 10,000×g for 10 min to remove the proteins. The oligomers in the samples were analyzed by thin layer chromatography (TLC). The solvent system was 1-butanol/acetic acid/water (4:6:1, v/v). The products were visualized by heating TLC plate at 90 °C for 15 min after spraying with 10% (v/v) sulfuric acid in ethanol. To determine the degree of polymerization (DP) of the oligomers in the samples, ESI-MS was used to determine the molecular mass of the oligomers in each sample on Agilent 6340 (Agilent, U.S.) with the conditions as following: capillary voltage, 1.8 kV; dry temperature, 325 °C, Gas, 4 L/min; scan range, 50~700 m/z.

## 3. Results

### 3.1. Screening and identification of strain Pseudoalteromonas sp. SM0524

**Figure 1:**
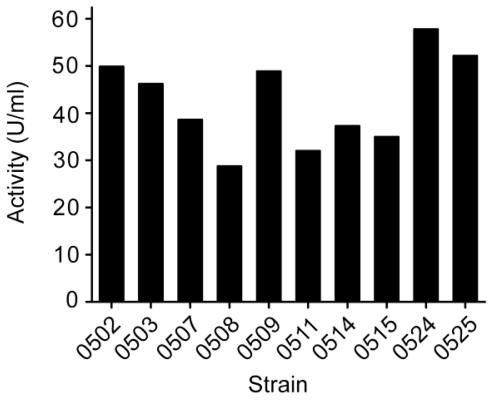
Alginate lyase activities in the culture of the ten selected strains. Each strain was inoculated into a liquid medium with the same composition as the enrichment medium and cultured at 180 rpm, 25 °C. After 44 h cultivation, the alginate lyase activity in the culture of each strain was measured.

Among the ten strains that were selected for their relatively bigger hydrolytic zones on the screening plates, strain 0524 had the highest rate to reduce the viscosity of the medium and the highest alginate lyase activity in its culture (Figure 1). This strain, named SM0524, was therefore selected for further study.

Strain SM0524 is a rod, Gram-negative bacterium. Colonies of strain SM0524 are pale-yellow round with orderly brim. Alignment of 16S rRNA gene sequences showed that strain SM0524 had 99% identity to many *Pseudoalteromonas* strains. Therefore, SM0524 is a strain of genus *Pseudoalteromonas*.

### 3.2. Purification of the alginate lyase aly-SJ02 from Pseudoalteromonas sp. SM0524

**Figure 2:**
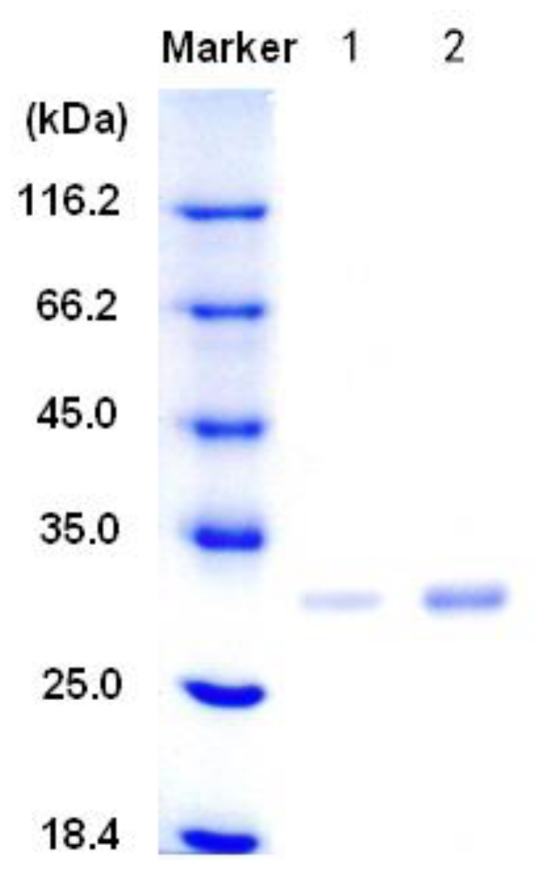
Purity and molecular mass of the alginate lyase aly-SJ02 SDS-PAGE analyzed by 10% SDS-PAGE. Lane 1: 5 μL of the purified aly-SJ02 (21 μg/mL); Lane 2: 25 μL of the purified aly-SJ02 (21 μg/mL).

After being cultured in the optimized medium for 60 h, the activity of alginate lyase from *Pseudoalteromonas* sp. SM0524 reached 62.6 ± 2.2 U/mL. The alginate lyase secreted by *Pseudoalteromonas* sp. SM0524 was purified by using ion-exchange and gel filtration chromatography as described in the Experimental Section (Figures S1–S3), which resulted in a 73-fold purification and a final yield of 45.1% (Table 1). The purity and molecular mass of the purified alginate lyase were analyzed by SDS-PAGE, which showed that the purified enzyme had an apparent molecular mass of 32 kDa (Figure 2). The purified alginate lyase from strain SM0524 was named aly-SJ02.

### 3.3. Characterization of the alginate lyase aly-SJ02 from Pseudoalteromonas sp. SM0524

**Figure 3:**
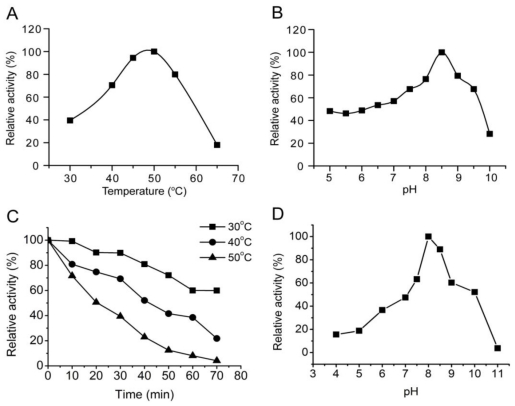
Effects of pH and temperature on the activity and stability of aly-SJ02 toward sodium alginate. (**A**) Effect of temperature on aly-SJ02 activity. The activity was assayed at pH 8.5. (**B**) Effect of pH on aly-SJ02 activity. The activity was measured at 50 °C in a broad buffer as described previously [9]. (**C**) Thermostability of aly-SJ02. The enzyme was incubated at 30 °C, 40 °C and 50 °C for different periods of time, and then the residual activity was assayed at 50 °C. (**D**) pH stability of aly-SJ02. Residual activities after incubation at various pHs for 20 min were assayed at pH 8.5, 50 °C. The data represent the mean of three experimental repeats with SD ≤ 5%.

**Figure 4:**
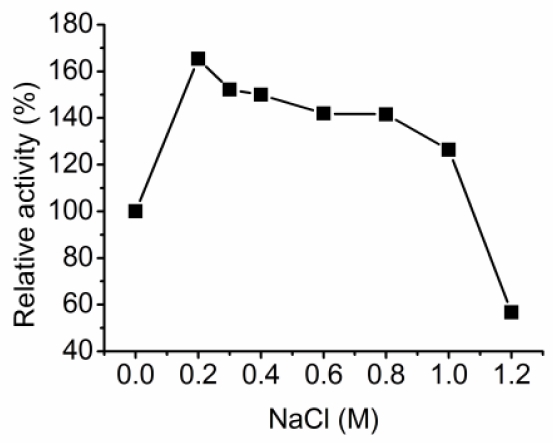
Effect of NaCl concentration on aly-SJ02 activity. The activity was measured at 40°C in 20 mM phosphate buffer containing the indicated NaCl concentrations. The activity in 0 M NaCl was taken as 100%. The data represent the mean of three experimental repeats with SD ≤ 5%.

With sodium alginate as substrate, aly-SJ02 displayed an optimal pH of 8.5 and an optimal temperature of 50 °C (Figure 3A, B). The half life period of aly-SJ02 was 41 min at 40 °C and 20 min at 50 °C, indicating that aly-SJ02 has low thermal stability (Figure 3C). Aly-SJ02 was most stable at pH 8.0 and retained more than 50% activity at pH 7.0–10 after incubation for 20 min (Figure 3D). The effects of metal ions on the activity of aly-SJ02 were analyzed. Most of the investigated metal ions, including Na^+^, K^+^, Mg^2+^, Ca^2+^, Co^2+^ Ba^2+^, Ni^2+^ and Sr^2+^, had activating effects on the activity of aly-SJ02. Zn^2+^ had no effect, and Cu^2+^ and Sn^2+^ had slight inhibitory effect on the activity of aly-SJ02. The presence of 1 mM EDTA decreased the enzyme activity to 48.3% of the control (Table 2), suggesting that aly-SJ02 may have metal ions in its structure. Aly-SJ02 showed the highest activity in 0.2 M NaCl, and retained more than 75% activity in 1 M NaCl, indicating its salt-tolerant ability (Figure 4).

Aly-SJ02 had activities toward both polyG and polyM, indicating that it is a bifunctional alginate lyase. It had almost the same activity toward sodium alginate and polyM, but lower activity toward polyG (Table 3). The *K*_m_ values of aly-SJ02 toward sodium alginate, polyA, and polyM at 50 °C, pH 8.5 were analyzed (Table 3). The result showed that the *K*_m_ value of aly-SJ02 toward polyG was the lowest, indicating that aly-SJ02 has the strongest affinity to polyG.

**Figure 5:**
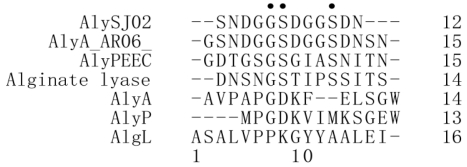
Alignment of aly-SJ02 to other alginate lyases. The dots above residues indicate the conserved amino acids. Sequences were aligned by using Clustal X 1.83 [12]. The figures on the right indicate the residue number of each sequence. alyA_AR06, an alginate lyase from *Pseudoalteromonas atlantica* AR06 (BAI50574.1, PL18); alyPEEC, an alginate lyase from *Pseudoalteromonas* sp. IAM14594 (AAD16034.1, PL18); alginatelyase, an alginate lyase from *Pseudoalteromonas* sp. 272 (1J1T_A, PL18); AlyA, an alginate lyase from *Klebsiella pneumoniae* subsp*. aerogenes* (AAA25049.1, PL7); AlyP, an alginate lyase from *Pseudomonas* sp. OS-ALG-9 (BAA01182.1, PL6); algL, an alginate lyase from *Azotobacter chroococcum* ATCC 4412 (CAA11481.1, PL5).

The N-terminal sequence of aly-SJ02 was analyzed by Edman degradation. The resultant sequence was SNDGGSDGGSDN. This sequence was blasted in NCBI database. The result showed that it had identity to some alginate lyases. The N-terminal sequence of aly-SJ02 was aligned with some alginate lyases from different polysaccharide lyase families (PL), which indicated that the N-terminal sequence of aly-SJ02 had higher identity to the alginate lyases in PL 18 than to those in other PL families (Figure 5). This suggests that aly-SJ02 may be an alginate lyase of PL 18.

### 3.4. Analysis of the oligomers released from polyM, polyG and sodium alginate by aly-SJ02

**Figure 6:**
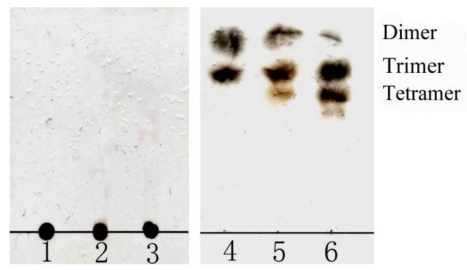
TLC analysis of the oligomers released from polyM, polyG and alginate by aly-SJ02. A 500 μL reaction mixture containing 1 μg aly-SJ02 and 2.5 mg polyM, polyG or sodium alginate was incubated at 40 °C for 2 h. Lane 1: sodium alginate; Lane 2: polyM; Lane 3: polyG; Lane 4: reaction products generated from sodium alginate. Lane 5: reaction products generated from polyM. Lane 6: reaction products generated from polyG. The DP of the oligomers in the products shown at the right of the figure was determined by ESI-MS.

**Figure 7:**
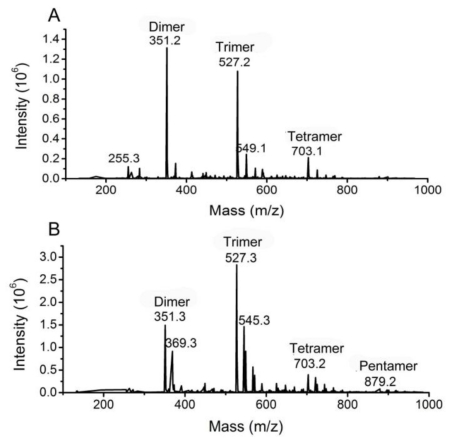
ESI-MS analysis of the degree of polymerization of the oligomers released from sodium alginate (**A**) and polyM (**B**) by aly-SJ02.

After polyG, polyM or sodium alginate was hydrolyzed by aly-SJ02 for 12 h, the released oligomers were analyzed by TLC. As shown in Figure 6, several kinds of oligomers were released from polyM, polyG and sodium alginate. Because there was no standard α-l-guluronic acid, β-d-mannuronic acid or their oligomers to determine the DP of the oligomers shown on TLC plate, ESI-MS was used to determine the DP of the oligomers. As shown in Figure 7, in polyM hydrolysate, M3 and M2 were the main products, and M4 only accounted for a very small fraction. The same case was shown in the hydrolysate of sodium alginate. That is, trimers and dimers were the main products, and tetramers accounted for a very small fraction, which was accordant with the result analyzed by TLC. On TLC plate, the tetramers in the hydrolysate of sodium alginate could not be detected, probably because their amount was too small. In order to confirm the results analyzed by ESI-MS, the peaks of dimers and trimers on ESI-MS were subjected to secondary mass spectrum. The results demonstrated that these peaks were dimers or trimers (Figure S5). The peaks of tetramers on ESI-MS were too small to be analyzed by secondary mass spectrum. Since polyG hydrolysate was precipitated by acetonitrile in preparing the sample for ESI-MS, the DP of the oligomers in polyG hydrolysate could not be analyzed by ESI-MS. However, by a comparison of the pattern of the oligomers in polyG hydrolysate and the pattern of the oligomers in the hydrolysates of polyM and sodium alginate shown on TLC plate (Figure 6), it could be deduced that trimers and tetramers were the main products in polyG hydrolysate, and the amount of dimers was very small.

## 4. Discussion

Bifunctional alginate lyases have been purified from some bacterial strains, such as *Alteromonas* sp. strain H-4 [13], *Alteromonas* sp. strain No. 272 [14] and *Pseudoalteromonas atlantica* AR06 [15]. These enzymes are different in molecular mass, stability, optimal pH and temperature, as well as the degree of polymerization of released oligosaccharides.

In this study, *Pseudoalteromonas* sp. SM0524 with high alginate lyase-producing ability was isolated from rotten kelp. In an optimized condition, the production of alginate lyase from *Pseudoalteromonas* sp. SM0524 reached 62.6 U/mL, suggesting that strain SM0524 is a good producer of alginate lyases. The alginate lyase secreted by *Pseudoalteromonas* sp. SM0524, aly-SJ02, was purified and characterized. Aly-SJ02 had an apparent molecular mass of 32 kDa. The optimal temperature and pH of aly-SJ02 toward sodium alginate was 50 °C and 8.5, respectively. Aly-SJ02 showed activities toward sodium alginate, polyG and polyM, indicating that it is a bifunctional alginate lyase. Alginate lyases are organized into PL 5, 6, 7, 14, 15, 17, and 18 (http://www.cazy.org/fam/acc_PL.html). The N-terminal sequence of aly-SJ02 has higher identity to the alginate lyases in PL 18 than to those in other PL, suggesting that aly-SJ02 may be an alginate lyase of PL 18.

The reported bifunctional alginate lyases release different oligosaccharides from polyG or polyM. The enzyme from *Alteromonas* sp. strain H-4 mainly released G6 and M5 [13]. The enzyme from *Alteromonas* sp. strain No. 272 mainly produced G3 and M3 [14]; and the main products released by the enzyme from *Pseudoalteromonas atlantica* AR06 were G3, G4, M3 and M4 [15]. In contrast to previously reported bifunctional alginate lyases, aly-SJ02 secreted by *Pseudoalteromonas* sp. SM0524 mainly released dimers and trimers from poly M and alginate, as well as G3 and G4 from polyG.

Depolymerized alginates have been shown to have wide applications. Some alginate-derived oligosaccharides had a growth-promoting effect on plants [16–18]. Oligomeric alginate can promote growth of *Bifidobacteria* spp. and thus has been proposed for use as a physiological food source [19,20]. Dimers, trimers, and tetramers, which possess guluronic acid at the reducing end, effectively induce the proliferation of keratinocytes in the presence of epidermal growth factor [21]. Some alginate polymers have antitumor effects [22], while others can enhance phagocytic activity of macrophages [23], and stimulate production of cytokines by human monocytes [24]. Alginate lyases are crucial in the generation of useful oligomeric products. Since the main oligomers released from alginate by aly-SJ02 are dimers and trimers, aly-SJ02 may be a good tool to produce these oligomers from alginate.

## 5. Conclusions

An alginate lyase-producing bacterial strain, *Pseudoalteromonas* sp. SM0524, was screened from marine rotten kelp. The alginate lyase aly-SJ02 secreted by strain SM0524 was purified by using ion-exchange and gel filtration chromatography. Aly-SJ02 had an apparent molecular mass of 32 kDa. The optimal temperature and pH of aly-SJ02 toward sodium alginate was 50 °C and 8.5, respectively. N-terminal sequence analysis suggested that aly-SJ02 may be an alginate lyase of PL 18. Aly-SJ02 is a bifunctional alginate lyase, which showed activities toward both polyG and polyM. Contrary to other bifunctional alginate lyases, Aly-SJ02 mainly released dimers and trimers from polyM and alginate, as well as G3 and G4 from polyG, suggesting that aly-SJ02 may be a good tool to produce dimers and trimers from alginate.

## Supplementary Data

Figure S1 Purification of aly-SJ02 by ion-exchange chromatography on a DEAE-Sepharose Fast Flow column.

Figure S2 SDS-PAGE analysis of the purity of the alginate lyase aly-SJ02 purified by ion-exchange chromatography. Lane 1–4: proteins in tube 22, 20, 18 and 16, respectively.

Figure S3 Purification of aly-SJ02 by gel filtration chromatography on a Sephadex G-100 column.

Figure S4 Non-linear fit curves for the hydrolysis of sodium alginate, polyG and polyM by aly-SJ-02. The initial rates were determined with 0–6.5 mg/mL of each substrate at 50 °C. The data represent the mean of three experimental repeats with SD ≤ 5%.

Figure S5 Secondary mass spectra of the dimers (**A**) and trimers (**B**) shown in Figure 6.

## Figures and Tables

**Table 1:** Purification of the alginate lyase secreted by *Pseudoalteromonas* sp. SM0524.

Purification step	Total volume (mL)	Total protein (mg)	Total activity (U)	Specific activity (U/mg)	Purification fold	Yield (%)
Culture supernatant	130	124.5	8139.3	65.4	1	100
Ion exchange	36	2.916	6536.5	2241.6	34.3	80.3
Gel filtration	15	0.765	3674.1	4802.7	73.4	45.1

**Table 2:** Effects of metal ions and EDTA on the activity of aly-SJ02 toward sodium alginate.

Metal ion (mM)	Relative activity a (%)	Metal ion/EDTA (mM)	Relative activity (%)
control	100	K^+^ (10)	117.0
Ba^2+^ (1)	143.6	Mn^2+^ (1)	115.6
Na^+^ (10)	143.4	Ni^2+^ (1)	110.5
Ca^2+^ (1)	136.0	Zn^2+^ (1)	100.6
Mg^2+^ (10)	125.7	Cu^2+^ (1)	98.9
Sr^2+^ (1)	124.1	Sn^2+^ (1)	95.3
Co^2+^ (1)	122.7	EDTA (1)	48.3

a The activity of aly-SJ02 without any metal ion or inhibitor in the reaction mixture was taken as control (100%). The data represent the mean of three experimental repeats with SD ≤ 5%.

**Table 3:** Specific activities and kinetic parameters of aly-SJ02 toward sodium alginate, polyM and polyG.

Substrate	sodium alginate	polyG	polyM
Specific activity^a^ (U/mg)	4802.7	3073.7	4153.8
*K*_m_^b^ (mg/mL)	1.086	0.465	2.751
*V*_max_^b^ (OD_235_/h)	8.074	5.318	7.131

a The activities of aly-SJ02 toward polyG and polyM were measured with the same method as that toward sodium alginate. The data represent the mean of three experimental repeats with SD ≤ 5%.

b The *K*_m_ and *V*_max_values were determined by non-linear fit analysis based on Michaelis-Menten equation as shown in Figure S4. The unit of the reaction velocity was defined as the increasing absorbance at 235 nm of the products generated by aly-SJ02 per hour.

## Abbreviations

G: α-l-guluronic acid
M: β-d-mannuronic acid
SDS-PAGE: sodium dodecyl sulfate-polyacrylamide gel electrophoresis
PL: polysaccharide lyase family
TLC: thin layer chromatography
