# Supplementary figures and images for: Purification and Characterization of a Bifunctional Alginate Lyase from Pseudoalteromonas sp. SM0524
Source: Mar Drugs. 2011 Jan 21;9(1):109–23. doi: 10.3390/md9010109 (PMC3039154; doi:10.3390/md9010109)

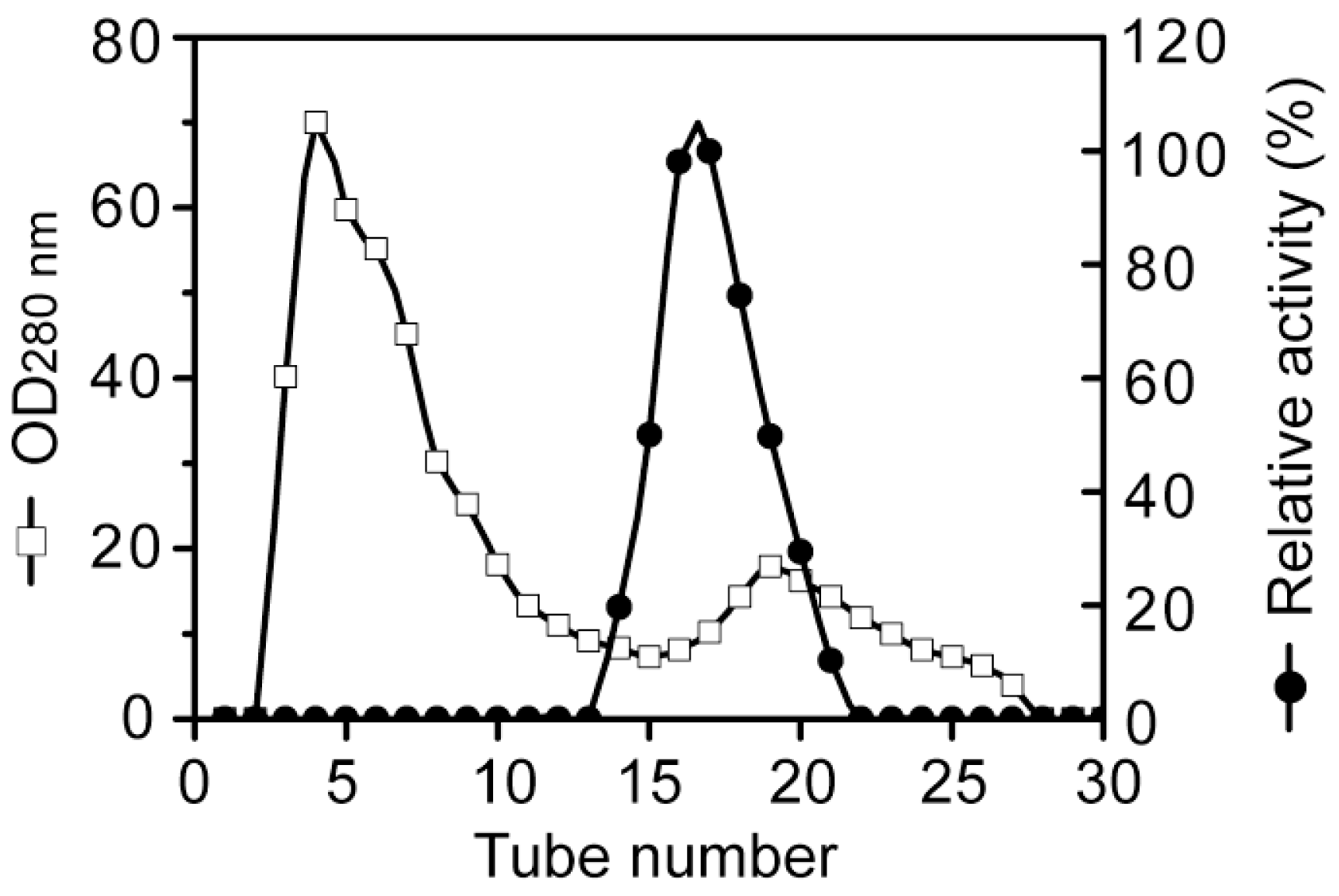

Supplement: Figure S1 — Purification of aly-SJ02 by ion-exchange chromatography on a DEAE-Sepharose Fast Flow column. [file marinedrugs-09-00109s1.tif]

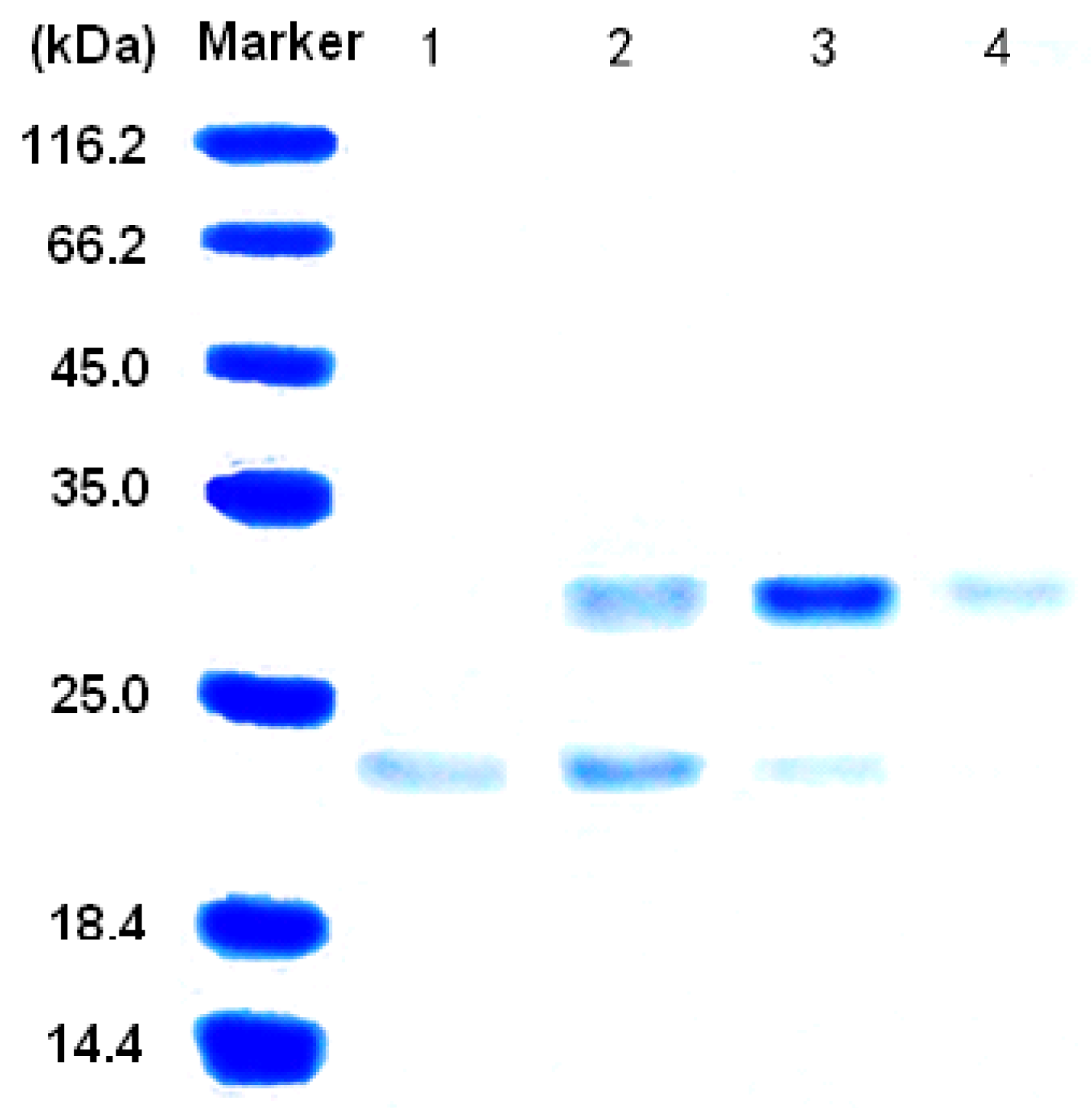

Supplement: Figure S2 — SDS-PAGE analysis of the purity of the alginate lyase aly-SJ02 purified by ion-exchange chromatography. Lane 1–4: proteins in tube 22, 20, 18 and 16, respectively. [file marinedrugs-09-00109s2.tif]

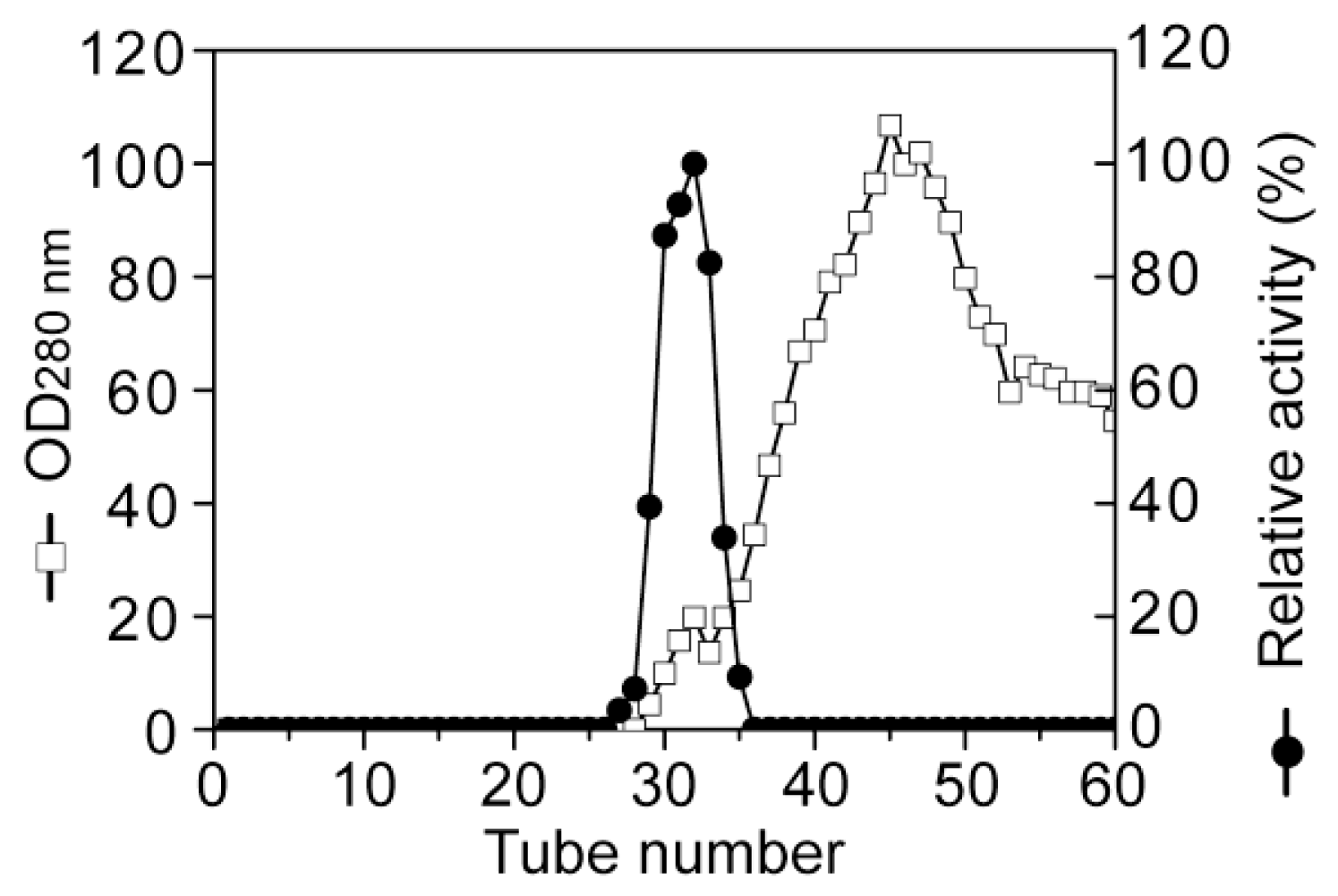

Supplement: Figure S3 — Purification of aly-SJ02 by gel filtration chromatography on a Sephadex G-100 column. [file marinedrugs-09-00109s3.tif]

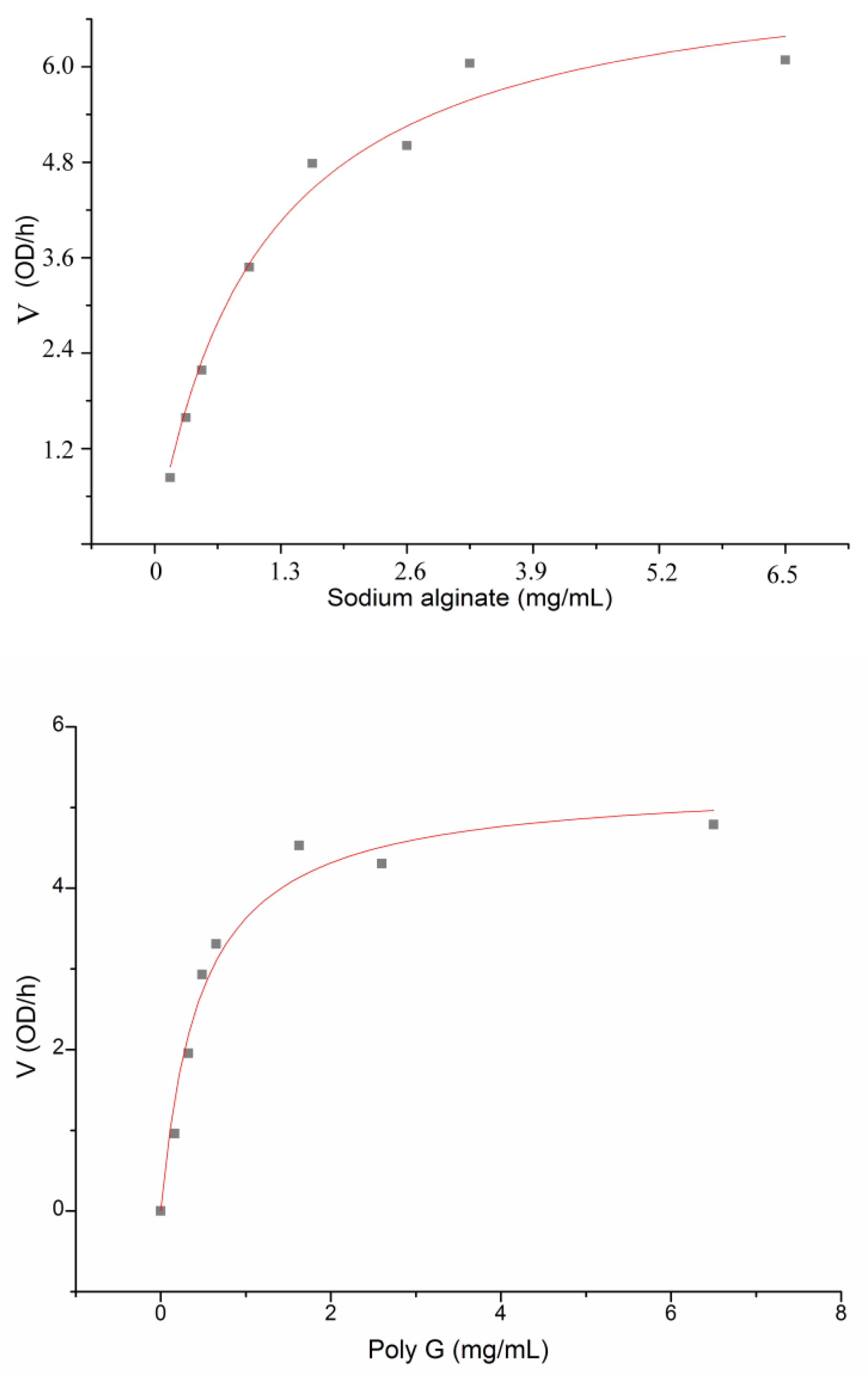

Supplement: Figure S4 — Non-linear fit curves for the hydrolysis of sodium alginate, polyG and polyM by aly-SJ-02. The initial rates were determined with 0–6.5 mg/mL of each substrate at 50 °C. The data represent the mean of three experimental repeats with SD ≤ 5%. [file marinedrugs-09-00109s4a.tif]

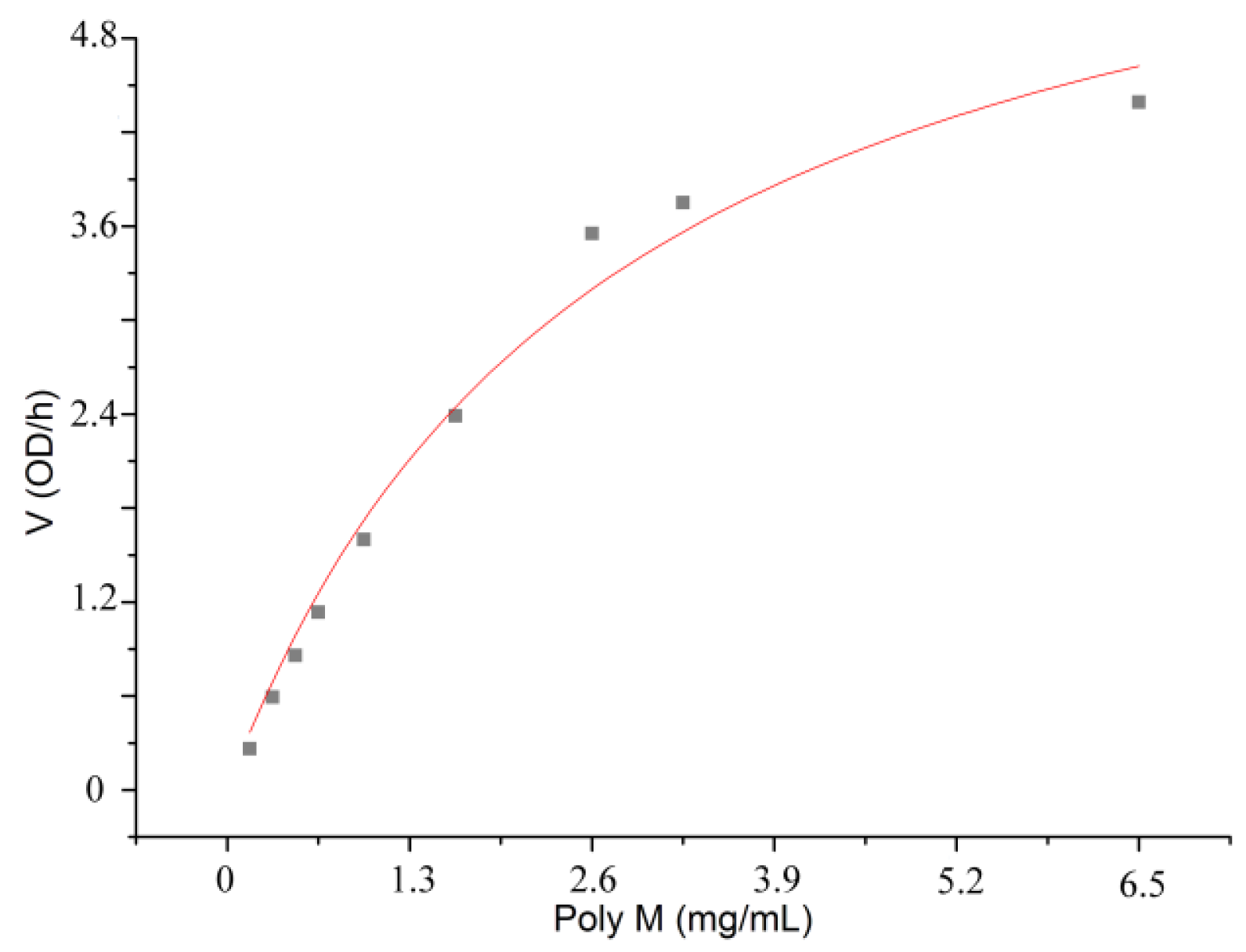

Supplement: Figure S4 — Non-linear fit curves for the hydrolysis of sodium alginate, polyG and polyM by aly-SJ-02. The initial rates were determined with 0–6.5 mg/mL of each substrate at 50 °C. The data represent the mean of three experimental repeats with SD ≤ 5%. [file marinedrugs-09-00109s4b.tif]

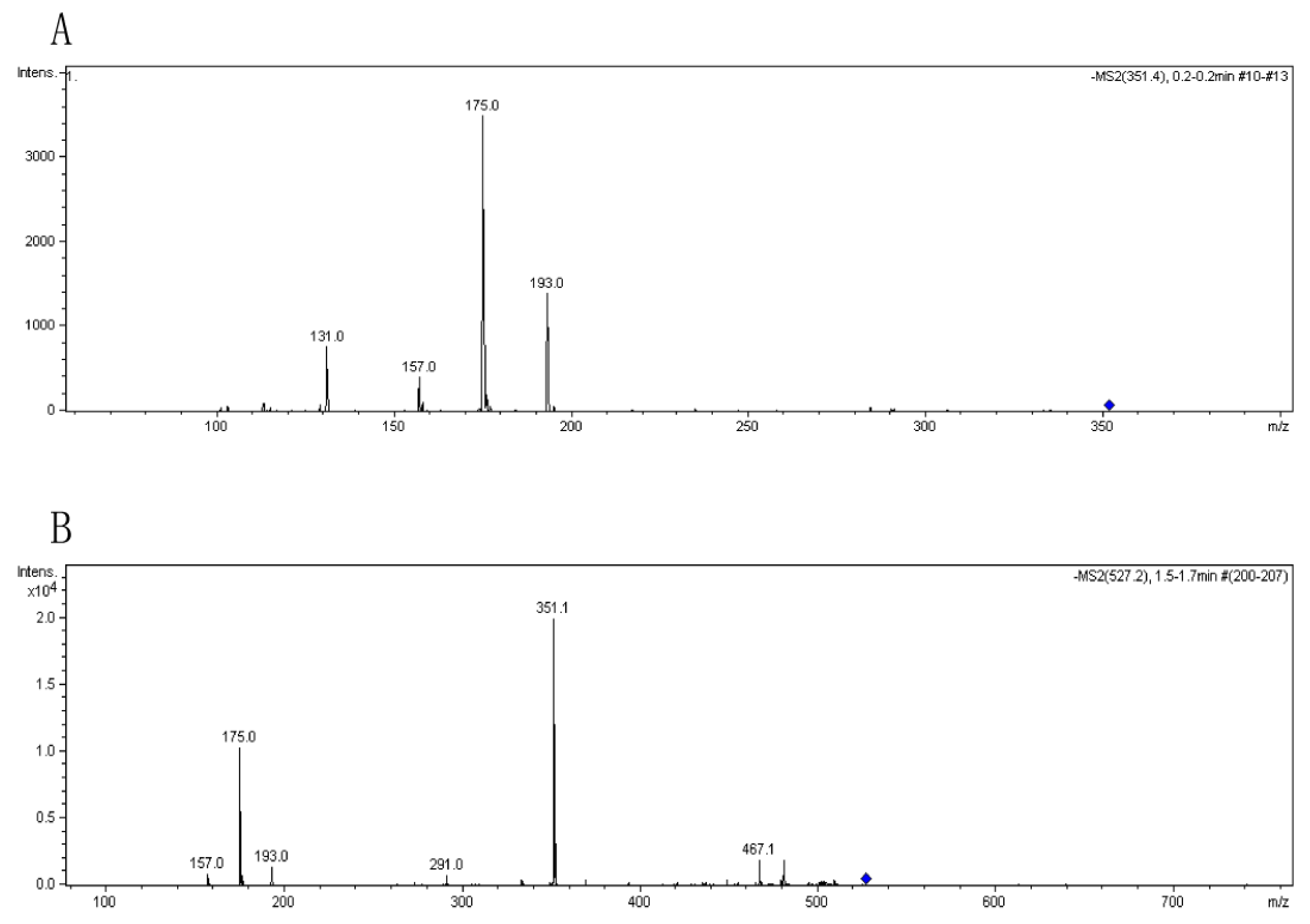

Supplement: Figure S5 — Secondary mass spectra of the dimers (A) and trimers (B) shown in Figure 6. [file marinedrugs-09-00109s5.tif]
